# Supplementary material for: A fault-intrusion-tolerant system and deadline-aware algorithm for scheduling scientific workflow in the cloud
Source: PeerJ Comput Sci. 2021 Nov 2;7:e747. doi: 10.7717/peerj-cs.747 (PMC8576554; doi:10.7717/peerj-cs.747)
Supplement: Supplemental Information 6 [file peerj-cs-07-747-s006.zip › Myproject1.1/docs/org/cloudbus/cloudsim/core/package-summary.html]

org.cloudbus.cloudsim.core


JavaScript is disabled on your browser.


- Overview
- Package
- Class
- Use
- Tree
- Deprecated
- Index
- Help

- Prev Package
- Next Package

- Frames
- No Frames

- All Classes

# Package org.cloudbus.cloudsim.core

- Class Summary

  | Class | Description |
  | --- | --- |
  |  |  |
  | --- | --- |
  | CloudInformationService | A Cloud Information Service (CIS) is an entity that provides cloud resource registration, indexing and discovery services. |
  | CloudSim | This class extends the CloudSimCore to enable network simulation in CloudSim. |
  | CloudSimShutdown | CloudimShutdown waits for termination of all CloudSim user entities to determine the end of simulation. |
  | CloudSimTags | Contains various static command tags that indicate a type of action that needs to be undertaken by CloudSim entities when they receive or send events. |
  | DeferredQueue | This class implements the deferred event queue used by `Simulation`. |
  | FutureQueue | This class implements the future event queue used by `Simulation`. |
  | SimEntity | This class represents a simulation entity. |
  | SimEvent | This class represents a simulation event which is passed between the entities in the simulation. |

- Overview
- Package
- Class
- Use
- Tree
- Deprecated
- Index
- Help

- Prev Package
- Next Package

- Frames
- No Frames

- All Classes
